# Affordable drug resistance genotyping of HIV-1 reverse transcriptase, protease and integrase genes, for resource limited settings

**DOI:** 10.1186/s12981-023-00505-3

**Published:** 2023-02-09

**Authors:** Sontaga Manyana, Melendhran Pillay, Lilishia Gounder, Aabida Khan, Pravi Moodley, Kogieleum Naidoo, Benjamin Chimukangara

**Affiliations:** 1 Department of Virology, School of Laboratory Medicine and Medical Sciences, University of KwaZulu-Natal and National Health Laboratory Service, 800 Vusi Mzimela Road, Durban, 4058 South Africa; 2 Centre for the AIDS Programme of Research in South Africa (CAPRISA), Durban, South Africa; 3 CAPRISA HIV-TB Pathogenesis and Treatment Research Unit, South African Medical Research Council (SAMRC), Durban, South Africa; 4 Critical Care Medicine Department, NIH Clinical Center, Bethesda, MD USA

**Keywords:** HIV-1, Pol gene, Affordable genotypic testing, Drug resistance, Resource limited settings, Primary integrase resistance

## Abstract

**Background:**

As use of dolutegravir (DTG) becomes more common in resource limited settings (RLS), the demand for integrase resistance testing is increasing. Affordable methods for genotyping all relevant HIV-1 *pol* genes (i.e., *protease* (PR*), reverse transcriptase* (RT) *and integrase* (IN)) are required to guide choice of future antiretroviral therapy (ART). We designed an in-house HIV-1 drug resistance (HIVDR) genotyping method that is affordable and suitable for use in RLS.

**Methods:**

We obtained remnant plasma samples from CAPRISA 103 study and amplified HIV-1 PR, RT and IN genes, using an innovative PCR assay. We validated the assay using remnant plasma samples from an external quality assessment (EQA) programme. We genotyped samples by Sanger sequencing and assessed HIVDR mutations using the Stanford HIV drug resistance database. We compared drug resistance mutations with previous genotypes and calculated method cost-estimates.

**Results:**

From 96 samples processed, we obtained sequence data for 78 (81%), of which 75 (96%) had a least one HIVDR mutation, with no major-IN mutations observed. Only one sample had an E157Q INSTI-accessory mutation. When compared to previous genotypes, 18/78 (23%) had at least one discordant mutation, but only 2/78 (3%) resulted in different phenotypic predictions that could affect choice of subsequent regimen. All CAPRISA 103 study sequences were HIV-1C as confirmed by phylogenetic analysis. Of the 7 EQA samples, 4 were HIV-1C, 2 were HIV-1D, and 1 was HIV-1A. Genotypic resistance data generated using the IDR method were 100% concordant with EQA panel results. Overall genotyping cost per sample was estimated at ~ US$43–$US49, with a processing time of ~ 2 working days.

**Conclusions:**

We successfully designed an in-house HIVDR method that is suitable for genotyping HIV-1 PR, RT and IN genes, at an affordable cost and shorter turnaround time. This HIVDR genotyping method accommodates changes in ART regimens and will help to guide HIV-1 treatment decisions in RLS.

**Supplementary Information:**

The online version contains supplementary material available at 10.1186/s12981-023-00505-3.

## Background

HIV-1 drug resistance (HIVDR) remains one of the greatest threats to achieving sustainable viral suppression using antiretroviral therapy (ART). HIVDR is largely driven by inadequate viral suppression in individuals on ART, resulting mainly from poor treatment adherence [1, 2]. Following ART scale-up, there have been global concerns over increasing levels of transmitted HIVDR [3]. Most studies have shown increasing levels of pre-treatment HIV-1 drug resistance (PDR) mainly driven by non-nucleoside reverse transcriptase inhibitor (NNRTI) mutations [4, 5], with a modelling study by Phillips et al., showing a benefit of changing first-line ART regimens in a setting of high NNRTI resistance [6]. This resulted in the World Health Organization (WHO) recommendation for use of dolutegravir (DTG) in all ART regimens [7], given DTG’s better tolerability, less adverse effects, higher genetic barrier to resistance, and availability in a fixed dose combination with tenofovir (TDF) and lamivudine (3TC), i.e. TLD.

Preliminary findings from NADIA and ARTIST trials have shown adequate viral suppression rates among individuals switching to second-line DTG-based ART, regardless of pre-existing resistance at 96-weeks and 48-weeks follow-up, respectively [8–10]. However, the ADVANCE study showed lower viral suppression rates among individuals on DTG-based ART at 96-weeks, raising concerns over long-term treatment outcomes in a setting with high NNRTI-PDR [11]. HIVDR mutations can be detected by genotypic testing to predict drug susceptibility [12], and understanding INSTI primary resistance prior to extensive DTG-rollout remains important. However, routine HIVDR testing is not available to the majority of people living with HIV in RLS due to the high costs of genotyping and the need for specialized facilities [13, 14]. The REVAMP study showed some benefit to genotypic resistance testing for failure on first-line NNRTI-based ART prior to drug selection, and a cost–benefit effect from the study is yet to be shown [15]. In South Africa, genotypic resistance testing is recommended at virologic failure on PI or INSTI-based regimens to inform ART drug selection [16].

With more studies reporting cases of DTG resistance [17–19] and with increased access to integrase strand transfer inhibitors (INSTIs), the demand for INSTI resistance testing has increased and there exists a risk of emergence of increasing INSTI resistance-associated mutations in RLS [20]. Most available HIVDR genotypic methods in RLS entail two separate assays for the detection of HIVDR mutations in the (i) *reverse transcriptase* (RT) and *protease* (PR) genes, and the (ii) *integrase* (IN) gene alone, such as the Applied Biosystems HIV-1 Genotyping kit [21]. In-house assays have been widely used for HIVDR genotyping especially in RLS where cost remains a major barrier. Reducing the cost of HIV-1 genotypic testing through affordable in-house assays and monitoring strategies could improve access [22], which will subsequently improve clinical decisions and treatment outcomes.

Therefore, we designed an affordable in-house HIV-1 drug resistance testing method for genotyping all relevant HIV-1 *pol* genes (i.e. PR, RT and IN) using a one-step reverse transcription polymerase chain reaction (PCR) and nested PCR on remnant plasma samples.

## Methods

We optimized reverse transcriptase and nested PCR for amplification of viral RNA from samples with viral loads (VLs) ≥ 1000 copies/mL (i.e. lower limit of detection); the VL used to determine ART failure in RLS [23]. We obtained stored remnant plasma samples from a CAPRISA 103 study (hereafter referred to as CAP103), which was a cross-sectional study aimed at determining prevalence of acquired drug resistance and subsequent susceptibility to DTG-based regimens, among ART-experienced individuals with virologic failure at the East Boom Community Health Care Center, in Pietermaritzburg, South Africa. Participants from CAP103 gave written informed consent for sample storage and for use of their stored samples in future studies. Sequencing in CAP103 was done using an Applied Biosystem HIV-1 Genotyping kit for the PR and RT genes only, with no IN sequencing in all except one participant. Details of the CAP103 study have been published previously [24]. Aliquots of the same samples were processed using the designed in-house method, hereafter referred to as the IDR method.

We also obtained 7 remnant samples from an external quality assessment (EQA) programme in which the National Health Laboratory Services (NHLS) Department of Virology participates annually to ensure laboratory proficiency testing and competence in HIVDR genotyping, according to ISO 15189 standards. The EQA panel received from the Quality Centre for Molecular Diagnostics (QCMD) consisted of plasma specimens with HIV VLs ≥ 1,000 copies/mL. The NHLS Department of Virology scored 100% in the HIVDR genotyping QCMD assessment. Aliquots of the same samples were genotyped and compared to EQA panel results for IDR method validation.

## Laboratory methods

### Viral RNA extraction and polymerase chain reaction

We retrieved remnant plasma samples from − 80 °C freezer and left them to equilibrate to room temperature prior to processing. In summary, we extracted viral RNA from 500 µl of plasma using a NucliSENS easyMAG automated extraction platform (bioMérieux, Marcy l’Etoile, France), according to manufacturer’s instructions. To increase amplification sensitivity, plasma samples can be spun at 23,000 × g for 1 h at 4 °C to pellet the virus prior to extraction, as described previously [25]. We eluted each viral RNA sample in 25 µl volume. We performed complimentary DNA synthesis and first-round PCR on a ProFlex PCR System (Applied Biosystems, Foster City, United States), to amplify an ~ 4 kb HIV-1 *pol* region using SuperScript IV One-Step RT-PCR System (Thermo Fisher Scientific, Waltham, US). Forward primer PANA2AF (GAGGCAATGAGCCAARCAAACA, HXB2: 1882–1903) and reverse primer PANA3AR (TTCCAGGGCTCTAGKTTAGG, HXB2: 5846–5865) were used in One-Step RT-PCR. For each sample, we added 5 µl of RNA for a total 25 µl reaction volume, and included a positive and negative control in each PCR. Details of PCR and amplification conditions are shown in Table 1.

**Table 1:** Reverse transcription and first-round PCR conditions

Reagent	Volume per reaction (µl)	Concentration per reaction
2X Reaction RT-PCR master mix	12.5	1X
Nuclease-free water	2.25	–
PANA2AF (5 µM)	2.5	0.5 µM
PANA3AR (5 µM)	2.5	0.5 µM
SSIV/platinum SuperFi DNA polymerase (2X)	0.25	0.02X
Total volume	20	–

Thermocycling conditions			
	Temperature (^o^C)	Time	Cycle(s)
cDNA synthesis	50	10 min	1
Pre-denaturation	98	2 min	1
Denaturation	98	10 s	40
Annealing	56	20 s	
Extension	72	2 min	
Final extension	72	10 min	1
Hold	4	∞	Hold

*cDNA* complimentary DNA; *RT-PCR* reverse transcription polymerase chain reaction; *SSIV* SuperScript IV enzyme; *µl* microliter; *µM* micromolar; ^*o*^*C* Degrees Celsius

We performed second-round nested PCR on a ProFlex PCR System (Applied Biosystems, Foster City, United States), using Platinum *Taq* DNA Polymerase (Thermo Fisher Scientific, Waltham, US). Forward primer Pro1 (TAGAGCCAACAGCCCCACCA, HXB2: 2147 -2166) and reverse primer 5066R (ATCATCACCTGCCATCT GTTTTCCAT, HXB2: 5041–5066) were used in the nested PCR. For each sample, we added 2 µl of first-round amplicon for a total 25 µl reaction volume. We verified successful amplification of an ~ 2.9 kb amplicon on 1% agarose gel. Details of second-round PCR and amplification conditions are shown in Table 2.

**Table 2:** Second-round PCR conditions

Second-round PCR Mastermix		
Reagent	Volume per reaction (µl)	Concentration per reaction
Nuclease-free Water	18.4	–
10X PCR Buffer	2.5	1X
MgCl_2_ (50 mM)	1.0	2 mM
dNTP (10 mM)	0.5	0.2 mM
Pro1 (5 µM)	0.25	0.05 µM
5066R (5 µM)	0.25	0.05 µM
Platinum *Taq* DNA Polymerase	0.1	–
Total volume	23	–

Thermocycling conditions			
	Temperature (^o^C)	Time	Cycle(s)
Pre-denaturation	94	2 min	1
Denaturation	95	10 s	40
Annealing	56	20 s	
Extension	72	2 min	
Final extension	72	10 min	1
Hold	4	∞	Hold

*dNTP* deoxynucleoside triphosphate; *MgCl*_*2*_ magnesium chloride; *mM* millimolar; *µl* microliter; *µM* micromolar; ^*o*^*C* Degrees Celsius

For any sample that failed amplification, we designed a two-fragment approach to amplify the PR, RT and IN genes separately. Details of primers, and PCR conditions for the two-fragment approach are shown in Additional file 1Tables S1–S4.

### PCR product purification and Sanger sequencing

For each successfully amplified sample, we performed PCR product purification by incubating 10 μl of amplicon with 4 μl of ExoSAP-IT Express PCR Product Cleanup reagent (Thermo Fisher Scientific, Waltham, US) at 37 °C for 4 min and 80 °C for 1 min, with a hold at 4 °C. We performed cycle sequencing using BigDye Terminator v3.1 kit (Applied Biosystems, Foster City, CA, US) and sequence reaction purification using BigDye XTerminator v3.1 purification kit (Applied Biosystems, Foster City, CA, US), as described previously [26]. We sequenced samples on an ABI 3730 Genetic Analyzer (Applied Biosystems, Foster City, United States) with 8 sequencing primers designed to cover complete HIV-1 PR, RT and IN genes. Four primers covered PR (codons 1 to 99) and RT (codons 1 to 560) genes, and four primers covered the IN gene (codons 1 to 288). Details of sequencing primers used are shown in Table 3. Primers used in this study were obtained from research articles published previously [26–28].

**Table 3:** Sequencing primers for complete HIV-1 PR, RT and IN sequencing

Primer (Direction)	Primer sequence	HXB2	Gene
RTC1F (Forward)	ACCTACACCTGTCAACATAATTG	2486–2508	PR and RT
RTC2R (Reverse)	TGTCAATGGCCATTGTTTAACCTTTGG	2630–2604	PR and RT
RTC3F (Forward)	CACCAGGGATTAGATATCAATATAATGTGC	2965–2994	PR and RT
RTC4R (Reverse)	CTAAATCAGATCCTACATACAAGTCATCC	3101–3129	PR and RT
KVL076 (Forward)	GCACAYAAAGGRATTGGAGGAAATGAAC	4161–4188	IN
KVL082 (Forward)	GGVATTCCCTACAATCCCCAAAG	4647–4669	IN
KVL083 (Reverse)	GAATACTGCCATTTGTACTGCTG	4750–4772	IN
PAN2R (Reverse)	CTGCCATCTGTTTTCCATAYTC	5037–5058	IN
Optional primers			
2586F (Forward)	AAGCCAGGAATGGATGGCCCA	2586–2606	PR and RT
2713R (Reverse)	GGATTTTCAGGCCCAATTTTTG	2713–2692	PR and RT
PAN3F (Forward)	TTAAAAGAAAAGGGGGGATTGGG	4783–4805	IN
KVL084 (Reverse)	TCCTGTATGCARACCCCAATATG	5243–5265	IN

*HXB2* nucleotide position of HIV-1 reference sequence; *IN* integrase; *PR* protease; *RT* reverse transcriptase

### Sequence analysis and phylogenetics

Following capillary electrophoresis, we performed sequence editing using Geneious Prime software 2021.1.1 (Biomatters Ltd, New Zealand) [29]. We assessed HIVDR mutations using the Stanford University HIV drug resistance database (version 9.0) [30]. We excluded sequences without complete PR and RT genes. Complete PR and RT gene sequence pairs were evaluated for differences between the IDR method and CAP103. We predicted subsequent ART regimens in discordant sequence pairs based on South African national ART guidelines and previous research evidence [16].

For phylogenetic analysis, we combined all sequence pairs and included HIV-1 reference sequences obtained from the Los Alamos Database (hiv.lanl.gov). We aligned sequences in Geneious software using ClustalW and trimmed sequences to compare similar gene regions between sequence pairs. We performed maximum likelihood tree reconstruction using a generalized time reversible model with proportion of invariable sites and gamma distribution (GTR + I + G), and with 100 bootstrap replicates.

For IDR method validation, we compared IDR sequence data to corresponding gene regions of EQA sequences generated at the NHLS Department of Virology. HIV-1 subtype classification of EQA sequences was determined from the Stanford HIV drug resistance database and confirmed using the REGA HIV-1 Subtyping Tool [31]. Detailed steps of the IDR method are available on protocols.io, dx.doi.org/. 10.17504/protocols.io.b5tvq6n6

### Cost-estimate analysis

We performed a cost breakdown of consumables required for the IDR method using pricing from product catalogues, and estimated genotyping turnaround time. At the time of analysis we used an exchange rate of $1 US Dollar to ZAR15 South African Rands. In addition, we estimated the cost of genotyping using the alternative two-fragment approach.

## Results

**Fig. 1:**
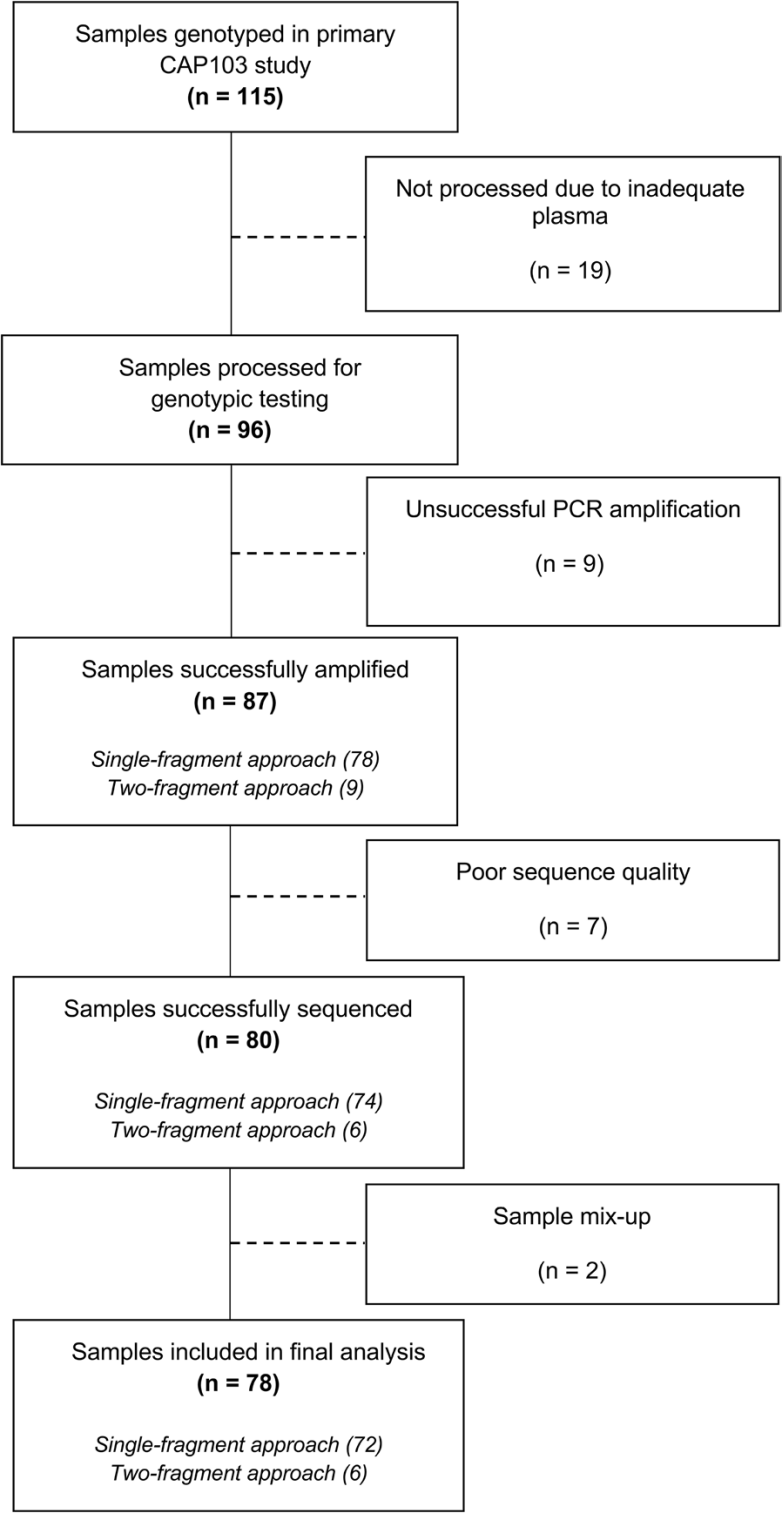
Summary flow chart of samples in IDR study from request to analysis

Overall, 115 remnant plasma samples with previous HIV-1 genotype results were obtained, and 19 samples were not processed due to inadequate plasma (i.e. plasma volume < 500 µl available for extraction). Of 96 samples processed, we included 78 (81%) samples with complete PR, RT and IN sequences in final analysis (Fig. 1). A complete list of all samples processed and their sequence outcomes are summarized in Additional file 1: Table S5.

### Amplification and sequencing

Overall, 87 of 96 (91%) samples were successfully amplified, 78 (90%) as a single amplicon of the PR, RT and IN genes, with an additional 9 samples amplified using the two-fragment approach (i.e. PR and RT, and a separate IN amplicon). The median.

**Fig. 2:**
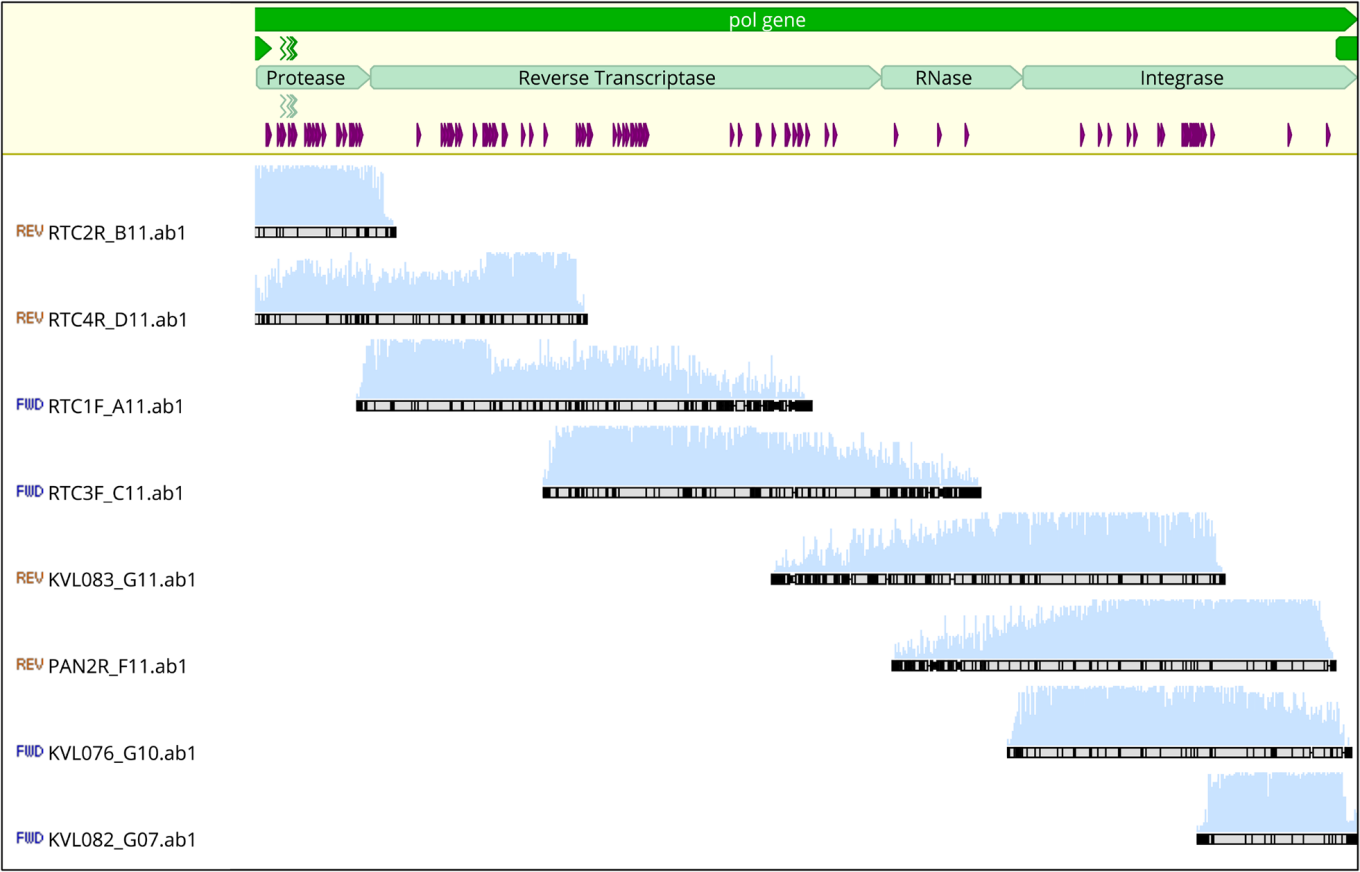
Complete sequence coverage of PR, RT and IN genes with 8 sequencing primers

VL of samples included in final analysis were higher compared to samples which failed amplification, i.e. median VL 4.4 log_10_ copies/mL, (interquartile range (IQR): 3.8 – 4.9) vs. 3.7 log_10_ copies/mL (IQR: 3.2–4.7), p = 0.06 (Wilcoxon rank-sum (Mann Whitney) test). A representative gel image of complete *pol* gene amplification is shown in Additional file 1: Figure S1. Sequence coverage of the complete *pol* gene was achieved using 8 sequencing primers as shown in Fig. 2.

### HIV drug resistance mutations and sequence comparison

Of 87 samples successfully sequenced, two (IDR074 and IDR076) were excluded due to potential sample mix-up, and 7 had poor sequence quality. Of 78 sequences included in final analysis, 75 (96%) had at least one drug resistance mutation in either PR and RT genes, similar to CAP103. There were no major IN drug resistance mutations observed. Only one sample (IDR113) had an IN accessory mutation (i.e. E157Q). We observed similar proportions of protease inhibitor (PI), nucleoside reverse transcriptase inhibitor (NRTI), and NNRTI resistance mutations when compared to previous CAP103 genotypes. The most common PI drug resistance mutation was M46I, occurring in 5/78 (6%) sequences. M184IV (66/78, 78%) and K65ENR (29/78, 37%) were the most common NRTI mutations detected, whilst K219EQR (23/78, 30%) was the most common thymidine analogue mutation (TAM) detected. The most common NNRTI mutations were K103NS (47/78, 60%) and V106AIMT (27/78, 35%).

Of the 75 sequence pairs with HIVDR mutations, 18 (23%) had at least one discordant mutation resulting in different phenotypic predictions (Additional file 1: Table S6). To assess true discordances between sequence pairs, chromatograms were reviewed at each discordant amino acid position by a second laboratory scientist to verify whether the discordances resulted from true mutation calls, or were a result of subjective calling of nucleotide bases. Notably, the majority (13/18) of sequence pair discordances were due to nucleotide mixtures, i.e. positions containing more than one nucleotide, with minor peak height being ≥ 25% of major peak height. However, only 2 of the discordant sequences were clinically significant, resulting in prediction of a different subsequent ART regimen, as shown in Table 4. Both participants were on efavirenz (EFV)-based first-line ART and had discordances in the NRTI and NNRTI mutations. Example chromatograms of discordances due to mixed bases in the two sequence pairs are shown in Additional file 1: Figure S2.

**Table 4:** Summary of discordant mutations affecting choice of subsequent ART regimen

		Protease mutations		Reverse transcriptase mutations		Discordant phenotypic predictions		Mutation score		Predicted regimen of choice	
IDR ID	Regimen	CAP103	IDR	CAP103	IDR	CAP103	IDR	CAP103	IDR	CAP103	IDR
036	TDF + XTC + EFV	None	None	**M41L**,D67N, **K70R**,M184V, K219Q V106M,V179D	**A62V**,**K65R,** D67N,M184V, K219Q V106M,V179D, **F227FL**	ABC: H AZT: **H** FTC: H 3TC: H TDF:** L** DOR:** I** EFV: H ETR: PL NVP: H RPV: L	ABC: H AZT: **S** FTC: H 3TC: H TDF:** H** DOR: **H** EFV: H ETR: PL NVP: H RPV: L	65 70 70 70 20 50 90 10 90 25	75 5 95 95 65 100 105 10 120 25	TDF + 3TC + DTG	AZT + 3TC + DTG
094	TDF + XTC + EFV	None	None	**A62AV,K65KR,**M184V,**K219KN** K103N,V106M	M184V K103N,V106M	ABC: **H** AZT: S FTC: H 3TC: H TDF: **H**	ABC:** L** AZT: S FTC: H 3TC: H TDF: **S**	70 -10 95 95 60	15 -10 60 60 -10	AZT + 3TC + DTG	TDF + 3TC + DTG

*3TC* lamivudine; *ABC* abacavir; *AZT* zidovudine; *DTG* dolutegravir; *EFV* efavirenz; *FTC* emtricitabine; *H* high-level resistance; *I* intermediate resistance; *L* low-level resistance; *PL* potential low-level resistance; *TDF* tenofovir; *S* susceptible; *XTC* lamivudine or emtricitabine

Bold values represent discordant results between CAP103 and IDR

### Phylogenetic analysis

**Fig. 3:**
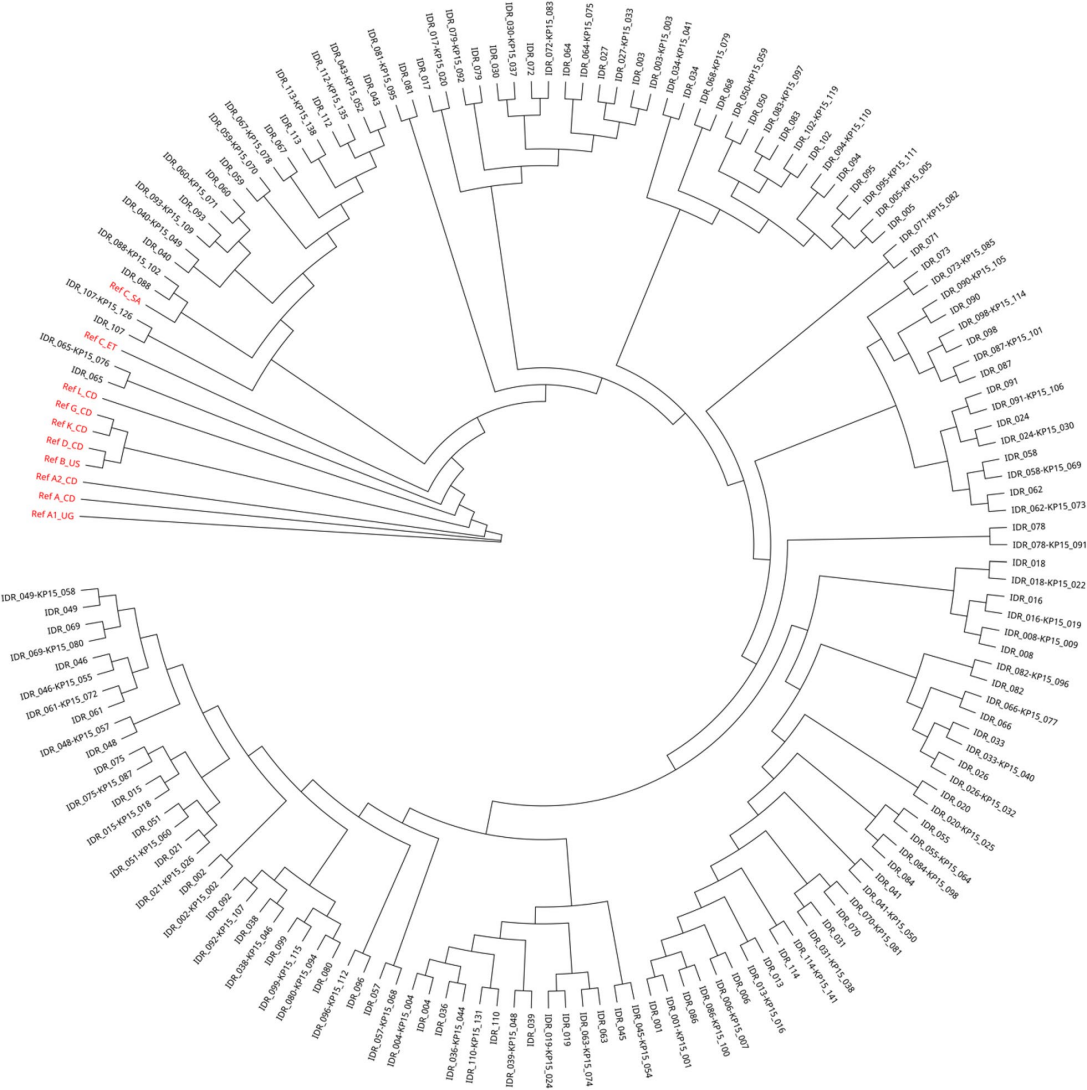
Maximum likelihood phylogenetic tree analysis of IDR and CAP103 sequence pairs

Phylogenetic tree reconstruction showed good concordance between sequence pairs of CAP103 and IDR study sequences, and all samples clustered around HIV-1 subtype C (HIV-1C) reference sequences, as shown in Fig. 3. Sequences generated from CAP103 study can be identified with a linked KP15 identification number.

### IDR method validation

Of 7 EQA samples, HIVDR mutations from IDR method showed 100% concordance with EQA panel results. Stanford HIVdb and REGA subtyping tool confirmed that sequences were from different HIV-1 subtypes, including subtypes A, C and D. Table 5 provides a summary of the EQA samples processed, their HIV-1 subtype classification, and specific mutations detected.

**Table 5:** Summary of EQA control samples processed for IDR method validation

Sample IDs	IDR IDs	Genes compared	HIV-1 subtype	Mutations
11.02A	EQA01	IN	A	IN: None
AA05694017	EQA02	PR and RT	C	PI: L10F,D30N,N88D NRTI: M41L,E44D,D67N,T69D,M184V,L210W,T215Y NNRTI: A98G
AA05694021	EQA03	PR and RT	D	PI: None NRTI: None NNRTI: None
AA05694023	EQA04	PR and RT	C	PI: M46I,I54V,V82A NRTI: M184V NNRTI: None
AA05694704	EQA05	PR, RT and IN	C	PI: None NRTI: D67N,K70R,M184V,K219Q NNRTI: None IN: None
AA05694734	EQA06	PR, RT and IN	D	PI: None NRTI: None NNRTI: None IN: None
AA05694741	EQA07	PR, RT and IN	C	PI: L10F,D30N,N88D NRTI: M41L,E44D,D67N,T69D,M184V,L210W,T215Y NNRTI: A98G IN: None

*EQA* external quality assessment; *IN* integrase; *NA* not applicable; *NRTI* nucleoside reverse transcriptase inhibitor; *NNRTI* non-nucleoside reverse transcriptase inhibitor; *PI* protease inhibitor; *PR* protease; *RT* reverse transcriptase

### Cost-estimate analysis

We estimated the cost of genotyping at ~ US$43–$US49 per sample, using the IDR single-fragment approach and alternative two-fragment approach, respectively (Additional file 1: Tables S7 and S8). The turnaround time required to genotype all relevant viral *pol* genes using the IDR method was estimated at ~ 15 h (~ 2 working days). Common genotyping methods (such as the Applied Biosystems HIV-1 Genotyping kit used for CAP103 genotyping) take approximately ~ 3 working days to genotype PR and RT genes only (Additional file 1: Figure S3).

## Discussion

As more countries in RLS roll-out DTG in first-line regimens as recommended by the WHO [7], the demand for INSTI resistance testing will substantially increase and a population level increase in INSTI resistant mutations is expected [22]. Common methods of HIVDR testing use an approach of genotyping the PR and RT genes separately from the IN gene. This is preferred because of more efficient amplification of shorter gene fragments. However, because of the need to genotype two separate fragments, such an approach doubles the workload and increases cost of genotyping, adding pressure on an already strained health care system. We designed a HIVDR method that effectively counteracts challenges associated with generating separate gene fragments. This simplifies the genotyping process, providing drug resistance profiles for all relevant viral genes at a low cost, with shorter turnaround times. These advantages over current methods of HIV-1 genotyping make the single-assay method ideal for use in RLS.

In this study we validate our method using 7 blinded remnant EQA samples from QCMD and apply the method to genotype remnant plasma samples from a previous CAPRISA study. QCMD is an international programme that offers quality assessment in molecular diagnostics to ensure laboratory proficiency testing and competence. Assessment of HIVDR genotyping is based on sequence alignment against all participants sequences submitted in the programme. We observed 100% concordance in detection of all drug resistance mutations, demonstrating the IDR method as reliable for HIVDR genotyping of the HIV-1 PI, RT and IN genes. With only 3/7 non-subtype C sequences (i.e. one HIV-1 A and two HIV-1D), further assessment of the method to non-subtype C sequences is warranted.

Using the IDR method, we did not detect any major primary INSTI resistance mutations. Only one of the 78 sequences had an INSTI accessory mutation E157Q. E157Q is a common polymorphic mutation observed in INSTI-naïve patients, with an estimated frequency of 0.5–2.3% across different subtypes [32–34]. When present alone, E157Q causes only potential low-level resistance to first-generation INSTIs (i.e. elvitegravir and raltegravir), with no resistance to DTG, cabotegravir and bictegravir [30]. However, it causes intermediate resistance to all INSTIs when it occurs together with R263K (a common INSTI mutation at ART failure), whilst decreasing DNA binding activity [35, 36]. As expected, we identified similar proportions of PI, NRTI and NNRTI drug resistance mutations as compared to previous CAP103 genotypes, with M46I, M184IV and K103NS being the most common mutations detected, respectively.

The two discordant sequences (IDR036 and IDR094) resulting in different choice of subsequent ART regimens had the M184V mutation which causes high-level resistance to 3TC and emtricitabine (FTC), whilst increasing viral susceptibility to zidovudine (AZT) and TDF [30]. Sequence IDR036 had multiple RT mutations, with discordances observed in both NRTI and NNRTI mutations. CAP103 detected TAMs M41L and K70R which were not detected in IDR, whilst IDR detected A62V and K65R mutations which were not detected in CAP103. K70R alone causes intermediate resistance to AZT, with M41L playing a minimal role in increasing AZT resistance in the absence of the T215Y mutation [37]. K65R detected by IDR causes high-level resistance to TDF and intermediate resistance to abacavir and 3TC/FTC, even in the absence of other NRTI mutations [30]. Addition of mutations A62V, D67N and K219Q (to K65R) results in high-level resistance to all NRTIs except AZT, thus a predicted AZT-based regimen would be recommended on the basis of the IDR sequence, whereas a predicted TDF-based regimen would be recommended on the basis of the CAP103 sequence. Detection of the mixture F227FL in IDR sequence had no significant impact on other NNRTIs and choice of DTG in subsequent ART regimen.

Sequence IDR094 had 3 discordant NRTI mutations, all of which were due to detection of nucleotide mixtures in CAP103 sequence. Detection of K65KR alone resulted in high-level TDF resistance thus a preferred AZT + 3TC + DTG subsequent regimen for CAP103, as opposed to TLD for IDR. Despite K219KN causing potential low-level resistance to AZT, presence of M184V reduced overall AZT resistance to susceptible. The reason for such discordances is not clear but could be explained by several reasons. Heterogenous distribution of HIV-1 variants in cells (arising from rapid evolution of HIV quasispecies), and absolute number of viral variants obtained during viral RNA extraction at any given time, could result in discordances associated with mixed bases [38]. Other factors to consider include, primer binding preference and location, general sequence quality, and technical errors introduced during PCR from *Taq* polymerase misincorporation [38].

Amplification and sequencing of larger gene regions by Sanger sequencing is met with challenges in obtaining successful PCR amplification and complete sequence coverage. In this study, we obtained ~ 91% (87/96) amplification success among samples with VLs ≥ 1,000 copies/mL (Fig. 1). Amplification success was improved by using the two-fragment approach which amplified an additional 9 samples. The 7 amplicons with failed sequencing had relatively lower band intensities observed in gel electrophoresis. The two sequences excluded from final analysis (IDR074 and IDR076) showed very high sequence similarity (> 98%) after repeating both samples from RNA extraction stage, with no evidence of epidemiological linkage. This suggested potential sample mix-up.

In efforts to provide genotyping results at the shortest time possible, this method reduces genotyping time from ~ 3 to ~ 2 days, saving at least one working day. In addition to providing timely results, it means more samples can be processed over time increasing the capacity of genotypic testing. We used Platinum *Taq* enzyme because it achieves full-length amplification of the ~ 2.9 kb *pol* gene of interest, without need for more expensive long-range enzymes. Also, we deliberately designed the method to use 8 sequencing primers, to make cycle sequencing reaction setup easier for laboratory operators working with standard 96-well plate formats. With this setup, sequencing primers are added in the 8 rows and samples in the 12 columns (Additional file 1: Figure S4), achieving coverage of all mutations of interest in the HIV-1 *pol* gene.

With cost remaining one of the major limiting factors to HIVDR genotyping in RLS [39], we estimated the cost per sample at ~ US$43–$US49 to genotype complete PR, RT, and IN genes in one fragment, or using the two-fragment approach (i.e., genotyping PR and RT separate from IN), respectively (Additional file 1: Tables S7 and S8). These estimates did not include labour and instrument maintenance costs, as well as assay accreditation/validation costs, which tend to vary by region. However, given that common in-house genotyping assays cost between US$48–US$155 to genotype the PR and RT genes only, with commercial assays ranging between US$155 and US$276 as described previously [39, 40], our method provides a cheaper option whilst genotyping not only the PR and RT genes, but also the IN gene.

There are some limitations to consider. Firstly, the majority of samples processed using the IDR method were HIV-1C samples, the most prevalent subtype accounting for almost half of all HIV infections globally and predominant in RLS [41]. However, we demonstrated successful sequencing of HIV-1A and HIV-1D subtypes in 3 of the 7 EQA samples processed. Secondly, prediction of subsequent ART regimens was based on levels of resistance and research evidence, but did not account for other clinical considerations that would typically guide treatment decisions, such as age, weight, co-infections (e.g. Hepatitis B status and tuberculosis), co-morbidities (e.g. renal impairment), and drug contraindications. Thirdly, we mostly compared sequence pairs in the PR and RT genes due to the parent (CAP103) study not having IN gene sequence data, although we would not expect a difference in concordance if paired IN sequences were included. Lastly, use of remnant samples meant that we could not process and compare ~ 17% of samples (19/115) due to low plasma volumes available for RNA extraction. Also, this potentially affected amplification success rates as RNA tends to degrade with repeated freeze thaw cycles.

In conclusion, we developed a simple, labour efficient and affordable HIVDR genotyping method for detecting mutations in the HIV-1 PR, RT and IN genes, and demonstrated high concordance with EQA samples. Despite discordances in two sequences resulting in differences in choice of subsequent regimens, recent data from NADIA trial (96-weeks follow up) showed TDF to be superior to AZT when administered with DTG, suggesting both patients would still benefit from switching to TLD. The lower cost, shorter turnaround time, coverage of all genes of interest, and ease of use, shows several advantages of this method over common in-house assays, making it ideal and relevant for use in monitoring HIVDR in RLS.

## Supplementary Information

**Additional file 1: Figure S1**. Gel image showing representation of amplicons after single-fragment PCR amplification. **Figure S2.** Comparison of discordant mutations between IDR and CAP103 sequences due to mixed bases. **Figure S3.** Comparison between IDR method and common in-house HIVDR genotyping workflows. **Figure S4.** Sample and sequencing primer layout in 96-well reaction plate. **Table S1**. PCR primers used to amplify PR, RT and IN genes using a two-fragment approach. **Table S2**. Reverse transcription and first-round PCR conditions for amplifying PR and RT genes with two-fragment approach. **Table S3**. Second-round PCR conditions for amplifying PR and RT genes with two-fragment approach. **Table S4**. One-step RT-PCR conditions for amplifying the IN gene with two-fragment approach. **Table S5**. Summary of all 96 samples processed and sequence outcome. **Table S6**. Details of discordant mutations resulting in discordant phenotypic predictions between IDR and CAP103 sequence pairs. **Table S7**. Approximate cost for genotypic testing using IDR single-fragment approach. **Table S8**. Approximate cost for genotypic testing using IDR two-fragment approach.

## Data Availability

Nucleotide sequence Accession numbers for IDR sequences and CAP103 sequences are available from GenBank Accession Numbers: OM468298–OM468467 and MW689343–MW689457, respectively.

## Abbreviations

3TC: Lamivudine
ART: Antiretroviral therapy
AZT: Zidovudine
CAPRISA: Centre for AIDS Programme Research in South Africa
DTG: Dolutegravir
EFV: Efavirenz
EQA: External quality assessment
FTC: Emtricitabine
HIVDR: HIV drug resistance
IN: Integrase
INSTI: Integrase strand transfer inhibitor
IQR: Interquartile range
NHLS: National Health Laboratory Services
NNRTI: Non-nucleoside reverse transcriptase inhibitor
NRTI: Nucleoside-reverse transcriptase inhibitor
PCR: Polymerase chain reaction
PDR: Pre-treatment HIV-1 drug resistance
PI: Protease inhibitor
PR: Protease
QCMD: Quality centre for molecular diagnostics
RLS: Resource limited setting
RT: Reverse transcriptase
TAM: Thymidine analogue mutation
TDF: Tenofovir
TLD: Tenofovir plus lamivudine and dolutegravir
TLE: Tenofovir plus lamivudine and efavirenz
VL: Viral load
WHO: World Health Organization
