# Supplementary material for: Affordable drug resistance genotyping of HIV-1 reverse transcriptase, protease and integrase genes, for resource limited settings
Source: AIDS Res Ther. 2023 Feb 9;20:9. doi: 10.1186/s12981-023-00505-3 (PMC9912687; doi:10.1186/s12981-023-00505-3)
Supplement: Supplementary file 1 — Additional file 1: Figure S1. Gel image showing representation of amplicons after single-fragment PCR amplification. Figure S2. Comparison of discordant mutations between IDR and CAP103 sequences due to mixed bases. Figure S3. Comparison between IDR method and common in-house HIVDR genotyping workflows. Figure S4. Sample and sequencing primer layout in 96-well reaction plate. Table S1. PCR primers used to amplify PR, RT and IN genes using a two-fragment approach. Table S2. Reverse transcription and first-round PCR conditions for amplifying PR and RT genes with two-fragment approach. Table S3. Second-round PCR conditions for amplifying PR and RT genes with two-fragment approach. Table S4. One-step RT-PCR conditions for amplifying the IN gene with two-fragment approach. Table S5. Summary of all 96 samples processed and sequence outcome. Table S6. Details of discordant mutations resulting in discordant phenotypic predictions between IDR and CAP103 sequence pairs. Table S7. Approximate cost for genotypic testing using IDR single-fragment approach. Table S8. Approximate cost for genotypic testing using IDR two-fragment approach. [file 12981_2023_505_MOESM1_ESM.pdf]

ADDITIONAL FILE

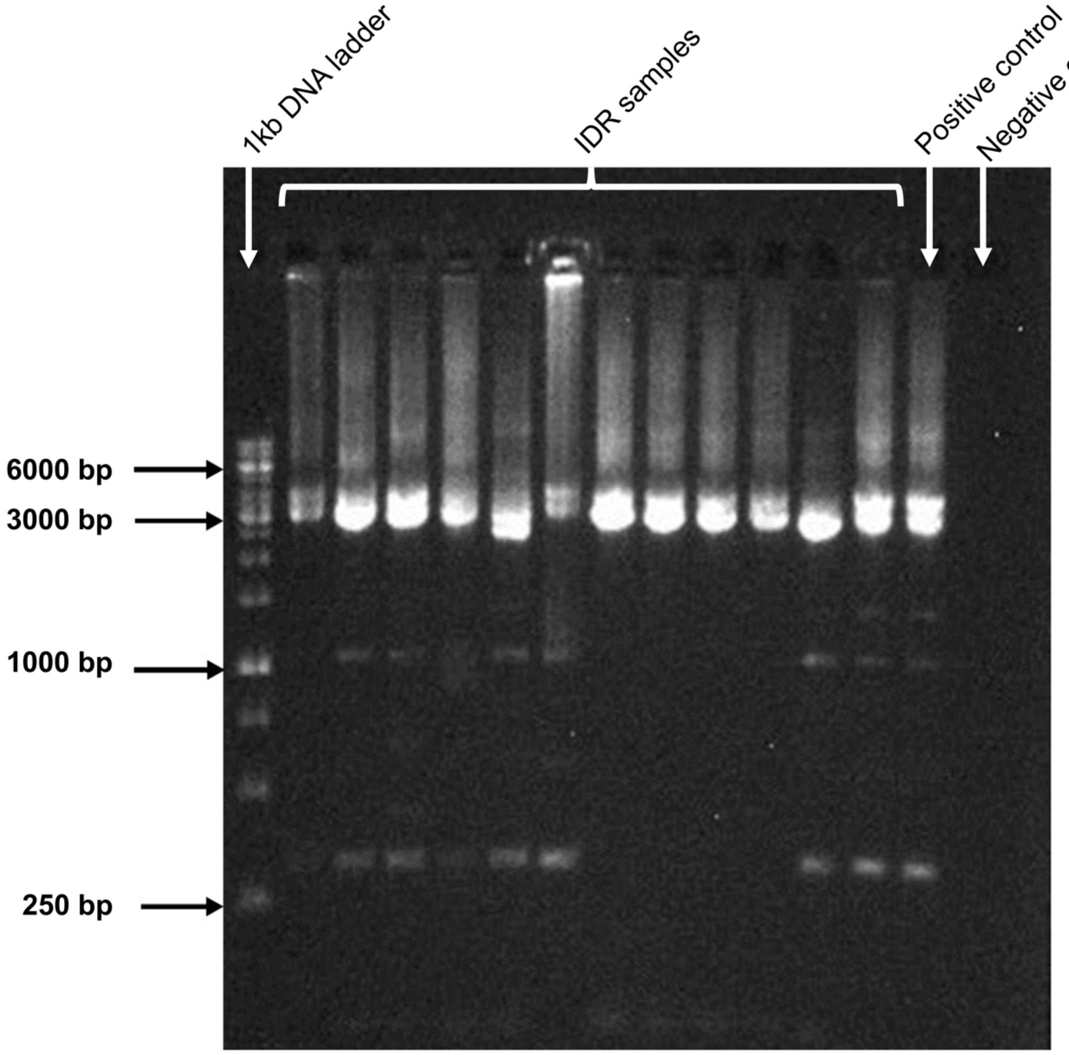

Figure S1 Gel image showing representation of amplicons after single-fragment PCR amplification

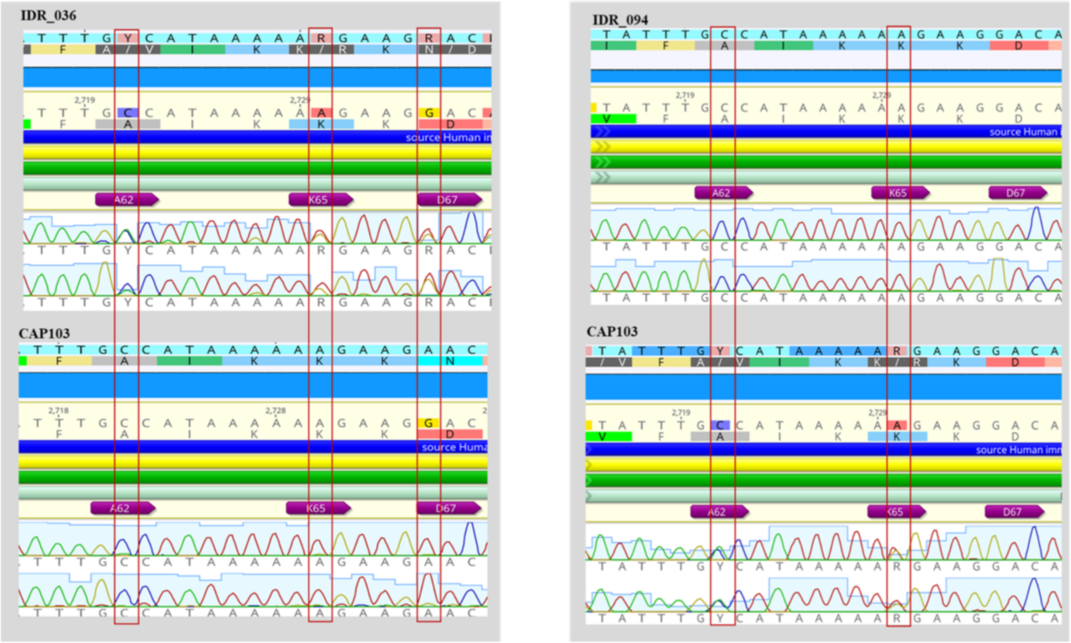

Figure S2 Comparison of discordant mutations between IDR and CAP103 sequences due to mixed bases

Note: In the first example (i.e. IDR036 on the left), the IDR sequence detected A62AV, K65KR and D67DN as mixtures that were not detected in CAP103 sequence. In the second example (i.e., IDR094 on the right), CAP103 sequence detected A62AV and K65KR mutations as mixtures that were not detected in the IDR sequence.

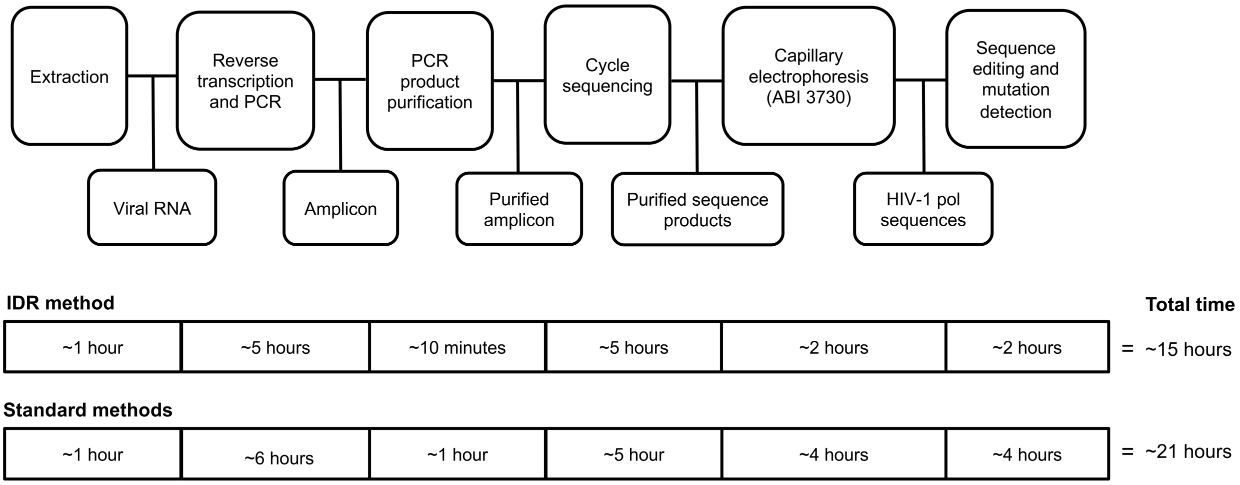

Figure S3 Comparison between IDR method and common in-house HIVDR genotyping workflows

	1	2	3	4	5	6	7	8	9	10	11	12
A	Sample 1_ RTC1F	Sample 2_ RTC1F	Sample 3_ RTC1F	Sample 4_ RTC1F				Primers				
B	Sample 1_ RTC2R	Sample 2_ RTC2R	Sample 3_ RTC2R	Sample 4_ RTC2R								
C	Sample 1_ RTC3F	Sample 2_ RTC3F	Sample 3_ RTC3F	Sample 4_ RTC3F								
D	Sample 1_ RTC4R	Sample 2_ RTC4R	Sample 3_ RTC4R	Sample 4_ RTC4R	Samples							
E	Sample 1_ KVL076	Sample 2_ KVL076	Sample 3_ KVL076	Sample 4_ KVL076								
F	Sample 1_ KVL082	Sample 2_ KVL082	Sample 3_ KVL082	Sample 4_ KVL082								
G	Sample 1_ KVL083	Sample 2_ KVL083	Sample 3_ KVL083	Sample 4_ KVL083								
H	Sample 1_ PAN2R	Sample 2_ PAN2R	Sample 3_ PAN2R	Sample 4_ PAN2R								

Figure S4 Sample and sequencing primer layout in 96-well reaction plate

Table S1 PCR primers used to amplify PR, RT and IN genes using a two-fragment approach

Primer (Direction)	Primer sequence	HXB2	Gene
MAW26 (Forward)	TCCCTCAGATCACTCTTTGGCAACGAC	2251 - 2277	PR and RT
RT21 (Reverse)	CTGTATTTCAGCTATCAAGTCCTTTGATGGG	3539 - 3509	PR and RT
Pro-1 (Forward)	TAGAGCCAACAGCCCCACCA	2147 - 2166	PR and RT
RT20 (Reverse)	CTGCCAATTCTAATTCTGCTTC	3462 - 3441	PR and RT
PANA3AF (Forward)	AGCATAGTAATATGGGGAAAGACTC	3684 - 3708	IN
5066R (Reverse)	ATCATCACCTGCCATCTGTTTTCCAT	5041 - 5066	IN

HXB2, nucleotide position of HIV-1 reference sequence; IN, integrase; PR, protease; RT, reverse transcriptase

Note: IN gene is amplified with only 2 primers using One-Step PCR

Table S2 Reverse transcription and first-round PCR conditions for amplifying PR and RT genes with two-fragment approach

**First-round PCR Mastermix**		
**Reagent**	**Volume per reaction (µl)**	**Concentration per reaction**
2X Reaction RT-PCR master mix	12.5	1X
Nuclease-free Water	2.25	-
MAW26 (5µM)	2.5	0.5µM
RT21 (5µM)	2.5	0.5µM
SSIV/ Platinum SuperFi DNA polymerase	0.25	0.02X
**Total volume**	**20**	-

**Thermocycling conditions**			
	**Temperature (°C)**	**Time**	**Cycle(s)**
cDNA synthesis	50	10 minutes	1
Pre-denaturation	98	2 minutes	1
Denaturation	98	10 seconds	
Annealing	56	20 seconds	40
Extension	72	2 minutes	
Final extension	72	10 minutes	1
Hold	4	∞	Hold

cDNA, complimentary DNA; RT-PCR, reverse transcription polymerase chain reaction; SSIV, SuperScript IV enzyme; µl, microliter; µM, micromolar; °C, Degrees Celsius

Table S3 Second-round PCR conditions for amplifying PR and RT genes with two-fragment approach

**Second round PCR Mastermix**			
**Reagent**		**Volume per reaction (µl)**	**Concentration per reaction**
Nuclease-free Water		18.4	-
10x PCR Buffer		2.5	1X
$MgCl_2$ (50mM)		1.0	2mM
dNTP (10mM)		0.5	0.2mM
Pro1 (5µM)		0.25	0.05µM
RT20 (5µM)		0.25	0.05µM
Platinum *Taq* DNA Polymerase		0.1	-
**Total volume**		**23**	-
**Thermocycling Conditions**			
	**Temperature (°C)**	**Time**	**Cycle(s)**
Pre-denaturation	94	2 minutes	1
Denaturation	95	10 seconds	
Annealing	56	20 seconds	40
Extension	72	2 minutes	
Final extension	72	10 minutes	1
Hold	4	∞	Hold

dNTP, deoxynucleoside triphosphate; $MgCl_2$, magnesium chloride; mM, millimolar; µl, microliter; µM, micromolar; °C, Degrees Celsius

Table S4 One-step RT-PCR conditions for amplifying the IN gene with two-fragment approach

**One-step PCR Mastermix**			
**Reagent**		**Volume per reaction (µl)**	**Concentration per reaction**
2X Reaction RT-PCR master mix		12.5	1X
Nuclease-free Water		1.5	-
PANA3AF (5µM)		2.5	0.5µM
5066R (5µM)		2.5	0.5µM
SSIV/ Platinum SuperFi DNA polymerase		1.0	0.08X
**Total volume**		**20**	-
**Thermocycling Conditions**			
	**Temperature (°C)**	**Time**	**Cycle(s)**
cDNA synthesis	50	10 minutes	1
Pre-denaturation	94	2 minutes	1
Denaturation	94	15 seconds	
Annealing	56	30 seconds	35
Extension	68	2 minutes	
Final extension	68	2 minutes	
Hold	4	∞	Hold

cDNA, complimentary DNA; RT-PCR, reverse transcription polymerase chain reaction; SSIV, SuperScript IV enzyme; µl, microliter; µM, micromolar; °C, Degrees Celsius

Table S5 Summary of all 96 samples processed and sequence outcome

IDR ID	Sex	Age	Log_{10} viral load copies/mL	Comments
IDR_001	Male	35	4,02	GSQ
IDR_002	Male	41	4,86	GSQ
IDR_003	Female	23	4,66	GSQ
IDR_004	Male	18	4,03	GSQ
IDR_005	Male	46	5,54	GSQ
IDR_006	Female	43	3,60	GSQ
IDR_008	Male	43	5,55	GSQ
IDR_013	Male	25	4,48	GSQ
IDR_014	Female	25	6,51	NAO
IDR_015	Male	47	3,88	GSQ
IDR_016	Female	39	3,31	GSQ
IDR_017	Male	20	4,45	GSQ
IDR_018	Female	36	4,40	GSQ
IDR_019	Female	30	3,81	GSQ
IDR_020	Female	30	4,17	GSQ
IDR_021	Female	30	4,54	GSQ
IDR_024	Male	53	4,20	GSQ
IDR_025	Female	33	3,16	NAO
IDR_026	Male	49	4,89	GSQ
IDR_027	Female	27	5,17	GSQ
IDR_028	Female	29	3,70	NAO
IDR_029	Male	40	3,17	NAO
IDR_030	Female	26	4,48	GSQ
IDR_031	Male	39	6,35	GSQ
IDR_033	Male	22	3,11	GSQ
IDR_034	Male	46	4,81	GSQ
IDR_036	Female	27	3,55	GSQ
IDR_038	Female	47	4,07	GSQ
IDR_039	Female	31	5,69	GSQ
IDR_040	Female	40	4,17	GSQ
IDR_041	Male	48	5,10	GSQ
IDR_043	Male	49	3,13	GSQ
IDR_045	Male	35	4,70	GSQ
IDR_046	Male	46	3,84	GSQ
IDR_048	Male	27	5,26	GSQ
IDR_049	Female	41	3,16	GSQ
IDR_050	Male	34	4,06	GSQ
IDR_051	Female	41	5,00	GSQ
IDR_055	Female	40	3,78	GSQ
IDR_056	Male	39	5,21	NAO
IDR_057	Male	67	5,86	GSQ
IDR_058	Female	46	3,41	GSQ
IDR_059	Female	21	5,36	GSQ
IDR_060	Male	51	5,59	GSQ
IDR_061	Male	37	4,37	GSQ
IDR_062	Male	33	3,91	GSQ
IDR_063	Male	30	3,45	GSQ
IDR_064	Male	36	4,80	GSQ
IDR_065	Female	35	4,46	GSQ
IDR_066	Female	47	5,09	GSQ

IDR_067	Male	46	4,07	GSQ
IDR_068	Female	41	4,82	GSQ
IDR_069	Male	43	4,74	GSQ
IDR_070	Female	36	4,33	GSQ
IDR_071	Male	32	4,77	GSQ
IDR_072	Female	41	3,45	GSQ
IDR_073	Female	25	3,80	GSQ
IDR_074	Female	52	4,74	Sample mix-up
IDR_075	Male	36	4,58	GSQ
IDR_076	Female	45	5,34	Sample mix-up
IDR_077	Male	50	3,05	NAO
IDR_078	Male	42	4,27	GSQ
IDR_079	Female	48	4,01	GSQ
IDR_080	Male	42	4,34	GSQ
IDR_081	Male	53	3,28	GSQ
IDR_082	Male	55	6,25	GSQ
IDR_083	Female	36	4,41	GSQ
IDR_084	Male	37	3,74	GSQ
IDR_085	Female	49	5,17	PSQ
IDR_086	Female	30	4,57	GSQ
IDR_087	Male	58	5,57	GSQ
IDR_088	Female	39	4,53	GSQ
IDR_089	Male	32	3,98	PSQ
IDR_090	Male	53	4,40	GSQ
IDR_091	Female	44	3,02	GSQ
IDR_092	Female	37	3,24	GSQ
IDR_093	Male	46	4,75	GSQ
IDR_094	Male	30	4,17	GSQ
IDR_095	Male	44	4,64	GSQ
IDR_096	Female	32	3,69	GSQ
IDR_097	Female	41	3,07	PSQ
IDR_098	Male	41	5,50	GSQ
IDR_099	Male	46	3,31	GSQ
IDR_100	Female	49	4,20	PSQ
IDR_101	Female	25	3,89	NAO
IDR_102	Female	23	5,43	GSQ
IDR_103	Female	32	3,20	NAO
IDR_104	Female	52	4,16	NAO
IDR_105	Male	20	5,27	PSQ
IDR_107	Male	29	6,22	GSQ
IDR_109	Male	35	4,01	PSQ
IDR_110	Female	38	5,53	GSQ
IDR_112	Female	37	3,91	GSQ
IDR_113	Male	44	6,62	GSQ
IDR_114	Female	47	3,45	GSQ
IDR_115	Female	30	6,28	PSQ

GSQ, good sequence quality; NAO, no amplification obtained; PSQ, poor sequence quality

Table S6 Details of discordant mutations resulting in discordant phenotypic predictions between IDR and CAP103 sequence pairs

		Protease		Reverse transcriptase		Discordant phenotypic predictions		Mutation score		Predicted regimen of choice	
**IDR ID**	**Line regimen**	**CAP103**	**IDR**	**CAP103**	**IDR**	**CAP103**	**IDR**	**CAP103**	**IDR**	**CAP103**	**IDR**
017	TDF+XTC+EFV	None	None	K65R,L74LI,<br>M184V	K65R,**K70KT**,<br>L74LI,M184V	ABC: H<br>AZT: S<br>FTC: H<br>3TC: H<br>TDF: **I**	ABC: H<br>AZT: S<br>FTC: H<br>3TC: H<br>TDF: **H**	105<br>-25<br>90<br>90<br>55	120<br>-25<br>100<br>100<br>80	AZT+3TC+DTG	AZT+3TC+DTG
020	AZT+XTC+LPVr	M46I,L76V	M46I,**I47IV**,<br>L76V	M184V<br>K103N,Y188L	M184V<br>K103N,Y188L	ATVr: **PL**<br>DRVr: **L**<br>LPVr: **I**	ATVr: **L**<br>DRVr: **I**<br>LPVr: **H**	10<br>20<br>50	20<br>30<br>65	TDF+3TC+DTG	TDF+3TC+DTG
021	TDF+XTC+EFV	None	None	**K70KEQ**,<br>M184V<br>K103N,V106M<br>E138A,V179L	M184V<br>K103N,V106M,<br>E138A,V179L	ABC: **I**<br>AZT: S<br>FTC: H<br>3TC: H<br>TDF: **L**	ABC: **L**<br>AZT: S<br>FTC: H<br>3TC: H<br>TDF: **S**	30<br>-20<br>70<br>70<br>15	15<br>-10<br>60<br>60<br>-10	TDF+3TC+DTG	TDF+3TC+DTG
036	TDF+XTC+EFV	None	None	**M41L**,D67N,**K70R**,<br>M184V,K219Q<br>V106M,V179D	**A62V**,**K65R**,<br>D67DN,M184V,<br>K219KQ<br>V106M,V179D,<br>**F227FL**	ABC: H<br>AZT: **H**<br>FTC: H<br>3TC: H<br>TDF: **L**<br>DOR: **I**<br>EFV: H<br>ETR: PL<br>NVP: H<br>RPV: L	ABC: H<br>AZT: **S**<br>FTC: H<br>3TC: H<br>TDF: **H**<br>DOR: **H**<br>EFV: H<br>ETR: PL<br>NVP: H<br>RPV: L	65<br>70<br>70<br>70<br>20<br>50<br>90<br>10<br>90<br>25	75<br>5<br>95<br>95<br>65<br>100<br>105<br>10<br>120<br>25	TDF+3TC+DTG	AZT+3TC+DTG
043	TDF+XTC+DTG<br>+LPVr+ETR	M46I<br>**L10LF**	M46I<br>None	**M41ML**,L210W,<br>T215F,K219R<br>K103N,Y181C,<br>P225H	M41L,L210W,<br>T215F,K219R<br>K103N,Y181C,<br>P225H	ATVr: PL<br>DRVr: **S**<br>LPVr: **L**	ATVr: PL<br>DRVr: **S**<br>LPVr: **PL**	10<br>5<br>15	10<br>0<br>10	TDF+3TC+DRV<br>+DTG	TDF+3TC+DRV+<br>DTG

045	AZT+XTC+LPVr	None	None	K65R,V75M,<br>M184V<br><br>K103N,G190S	K65R,V75M,<br>M184V<br><br>**L100LI**,K103N,<br>G190S	DRVr: **I**<br>EFV: H<br>ETR: **PL**<br>NVP: H<br>RPV: L	DRVr: **H**<br>EFV: H<br>ETR: **I**<br>NVP: H<br>RPV: H	30<br>120<br>10<br>120<br>15	60<br>180<br>40<br>180<br>75	TDF+3TC+DTG	TDF+3TC+DTG
048	TDF+XTC+EFV	None	None	K103N,G190GA<br><br><br>None	K103KN,<br>**V106VM,**G190A<br><br>None	DOR: **S**<br>EFV: H<br>ETR: PL<br>NVP: H<br>RPV: L	DOR: **I**<br>EFV: H<br>ETR: PL<br>NVP: H<br>RPV: L	0<br>105<br>10<br>120<br>15	50<br>165<br>10<br>180<br>15	TDF+3TC+DTG	TDF+3TC+DTG
062	TDF+XTC+EFV	None	**M46L**	K65R,M184V,<br>K219E<br><br>L100I,K103N,<br>M230L	K65R,M184V,<br>K219E<br><br>L100I,K103N,<br>M230L	ATVr: **S**<br>DRVr: S<br>LPVr: **S**	ATVr: **PL**<br>DRVr: S<br>LPVr: **PL**	0<br>0<br>0	10<br>0<br>10	AZT+3TC+DTG	AZT+3TC+DTG
067	TDF+XTC+EFV	None	None	**D67DN**,**L74LI**,<br>M184V<br><br>K103N,V106M	**D67G,K70E,**<br>M184V<br><br>K103N,V106M	ABC: **H**<br>AZT: S<br>FTC: H<br>3TC: H<br>TDF: **S**	ABC: **I**<br>AZT: S<br>FTC: H<br>3TC: H<br>TDF: **L**	65<br>5<br>60<br>60<br>0	35<br>-10<br>70<br>70<br>20	TDF+3TC+DTG	TDF+3TC+DTG
079	AZT+XTC+LPVr	None	None	M184V<br><br><br>**A98AG**,K103N,<br>**V108VI**,P225H	**M41ML**,M184V<br><br><br>K103N,P225H	ABC: L<br>AZT: S<br>FTC: H<br>3TC: H<br>TDF: S<br>DOR: **H**<br>EFV: H<br>ETR: **PL**<br>NVP: H<br>RPV: **L**	ABC: L<br>AZT: S<br>FTC: H<br>3TC: H<br>TDF: S<br>DOR: **I**<br>EFV: H<br>ETR: **S**<br>NVP: H<br>RPV:**S**	15<br>-10<br>60<br>60<br>-10<br>60<br>130<br>10<br>150<br>15	20<br>5<br>60<br>60<br>-5<br>30<br>105<br>0<br>105<br>0	TDF+3TC+DTG	TDF+3TC+DTG

080	AZT+XTC+LPVr	None	None	M184V	M184V	DOR: **L**	DOR: **S**	20	0	TDF+3TC+DTG	TDF+3TC+DTG
						EFV: H	EFV: H	120	90		
				**K101KE**,**K103KN**,	**K103S,**G190A	ETR: **I**	ETR: **PL**	30	10		
				G190A		NVP: H	NVP: H	150	120		
						RPV: **H**	RPV: **L**	60	15		
084	TDF+XTC+EFV	None	None	D67N,T69TADN,	D67N,K70R,	ABC: H	ABC: H	70	60	TDF+3TC+DTG	TDF+3TC+DTG
				**K70KEGR**,M184V,	M184V,K219Q	AZT: I	AZT: I	55	55		
				**K219KQR**		FTC: H	FTC: H	80	70		
						3TC: H	3TC: H	80	70		
						TDF: **I**	TDF: **L**	35	15		
				K103N,H221HY,	K103N,P225H,	DOR: I	DOR: I	45	30		
				**P225HR**,K238T	K238T	EFV: H	EFV: H	145	135		
						ETR: **PL**	ETR: **S**	10	0		
						NVP: H	NVP: H	150	135		
						RPV: **L**	RPV:**S**	15	0		
092	TDF+XTC+EFV	None	None	**D67DN**,M184V,	D67N,M184V,	ABC: L	ABC: L	25	25	TDF+3TC+DTG	TDF+3TC+DTG
				K219R	K219R	AZT: L	AZT: L	15	15		
						FTC: H	FTC: H	60	60		
				K103N,V108I,	K103N,V108I	3TC: H	3TC: H	60	60		
				**F227FL**		TDF: S	TDF: S	0	0		
						DOR: **H**	DOR: **L**	65	15		
						EFV: H	EFV: H	85	70		
						ETR: S	ETR: S	0	0		
						NVP: H	NVP: H	105	75		
						RPV:S	RPV:S	0	0		
093	AZT+XTC+LPVr	None	None	L74I,Y115F,	L74I,Y115F,	ABC: H	ABC: H	120	125	TDF+3TC+DTG	TDF+3TC+DTG
				M184V	M184V,**K219KE**	AZT: S	AZT: S	-10	0		
						FTC: H	FTC: H	60	60		
				V106M,H221Y,	V106M,H221HY,	3TC: H	3TC: H	60	60		
				F227C	F227C	TDF: **PL**	TDF: **L**	10	15		

094	TDF+XTC+EFV	None	None	**A62AV,K65KR,**	M184V	ABC: **H**	ABC: **L**	70	15	AZT+3TC+DTG	TDF+3TC+DTG
				M184V,**K219KN**		AZT: S	AZT: S	-10	-10		
						FTC: H	FTC: H	95	60		
				K103N,V106M	K103N,V106M	3TC: H	3TC: H	95	60		
						TDF: **H**	TDF: **S**	60	-10		
095	AZT+XTC+LPVr	None	None	M184V	**K70KE,**M184V	ABC: **L**	ABC: **I**	15	30	TDF+3TC+DTG	TDF+3TC+DTG
						AZT: S	AZT: S	-10	-20		
						FTC: H	FTC: H	60	70		
				K103N,H221Y,	K103N,H221Y,	3TC: H	3TC: H	60	70		
				M230L	M230L	TDF: **S**	TDF: **L**	-10	15		
096	TDF+XTC+EFV	None	None	D67N,K70R,	D67N,K70R,	ABC: H	ABC: H	70	60	TDF+3TC+DTG	TDF+3TC+DTG
				**L74I**,M184V	M184V**,K219Q**	AZT: I	AZT: I	35	55		
						FTC: H	FTC: H	60	70		
				K103N,V106M	K103N,V106M	3TC: H	3TC: H	60	70		
						TDF: **S**	TDF: **L**	5	15		
099	AZT+XTC+LPVr	None	None	**A98AG**	None	DOR: **L**	DOR: **S**	15	0	TDF+3TC+DTG	TDF+3TC+DTG
						EFV: **L**	EFV: **S**	15	0		
				M184V	M184V	ETR: **PL**	ETR: **S**	10	0		
						NVP: **I**	NVP: **S**	30	0		
						RPV: **L**	RPV: **S**	15	0		

3TC, lamivudine; ABC, abacavir; ATV, atazanavir; AZT, zidovudine; DOR, doravirine; DRVr, ritonavir-boosted darunavir; DTG, dolutegravir; EFV, efavirenz; ETR, etravirine; FTC, emtricitabine; H, high-level resistance; **I**, intermediate resistance; L, low-level resistance; LPVr, ritonavir-boosted lopinavir; NVP, nevirapine; PL, potential low-level resistance; RPV, rilpivirine; TDF, tenofovir; S, susceptible; XTC, lamivudine or emtricitabine

Table S7 Approximate cost for genotypic testing using IDR single-fragment approach

	Price ($US)	Number of samples	Price per sample ($US)	Total used	Total cost ($US)
**RNA Extraction**					
NucliSENS EasyMAG Kit	2598,70	1000	2,60	1	2,60
**PCR**					
SuperScript IV One-Step RT-PCR System (50µl)	824,27	200	4,12	1	4,12
Platinum Taq DNA Polymerase (120 reactions)	105,83	240	0,44	1	0,44
10mM dNTP Mix (0.2mL)	19,01	400	0,05	1	0,05
PCR Primers	13,33	700	0,02	2	0,04
Nuclease-Free Water (500mL)	46,82	3400	0,01	1	0,01
**Gel electrophoresis**					
TopVision Agarose Tablets (500mg x 200)	84,71	1000	0,08	1	0,08
UltraPure TBE Buffer, 10X (1L)	17,66	1000	0,02	1	0,02
SYBR Safe DNA Gel Stain (400µl)	70,27	400	0,18	1	0,18
GeneRuler 1kb DNA Ladder (2mL)	42,01	3300	0,01	1	0,01
6X Orange DNA Loading Dye (5mL)	28,51	330	0,09	1	0,09
**PCR product purification**					
ExoSAP-IT Express PCR Product Cleanup Reagent	237,90	480	0,50	1	0,50
**Sequencing and analysis**					
BigDye Terminator v3.1 Cycle Sequencing Kit (100 reactions)	1168,20	250	0,58	1	4,67
BigDye XTerminator Purification Kit (1000 reactions)	1346,40	250	0,67	1	5,39
POP-7 Polymer (25mL)	1578,60	8000	0,20	8	1,58
3730 Running Buffer, 10X (500mL)	232,50	200	1,16	1	1,16
Sequencing Primers	13,33	11250	0,00	8	0,01
**Consumables**					
1000µl tips (960)	132,09	960	0,14	6	0,83
200µl tips (960)	168,21	960	0,18	31	5,43
20µl tips (960)	112,14	960	0,12	5	0,58
10µl tips (960)	168,28	960	0,18	46	8,06

MicroAmp 8-Tube Strip with Caps, 0.2 mL	101,96	1000	0,10	3	0,31
1.5mL Nuclease-Free Eppendorf Tubes	63,13	500	0,13	5	0,63
50mL Falcon Tubes	132,47	25	5,30	1	5,30
MicroAmp Optical 96-Well Reaction Plates (10)	56,13	960	0,06	8	0,47
Adhesive PCR Plate Foils (100)	69,59	1000	0,07	1	0,07
**Total**					**$US42,62**

dNTP, deoxynucleoside triphosphate; g, gram; kb, kilobase; mL, millilitre; mM, millimolar; PCR, polymerase chain reaction; µl, microliter; µM, micromolar; $US, United States Dollar

Note: The exchange rate used was ~$1 US Dollar equivalent to ZAR15 South African Rands

Table S8 Approximate cost for genotypic testing using IDR two-fragment approach

	Price ($US)	Number of samples	Price per sample ($US)	Total used	Total cost ($US)
**RNA Extraction**					
NucliSENS EasyMAG Kit	2598,70	1000	2,60	1	2,60
**PCR**					
SuperScript IV One-Step RT-PCR System (50µl)	824,27	150	5,50	1	5,50
Platinum Taq DNA Polymerase (120 reactions)	105,83	240	0,44	1	0,44
10mM dNTP Mix (0.2 mL)	19,01	400	0,05	1	0,05
PCR Primers	13,33	700	0,02	2	0,04
Nuclease-Free Water (500mL)	46,82	3300	0,01	1	0,01
**Gel electrophoresis**					
TopVision Agarose Tablets (500mg x 200)	84,71	1000	0,08	1	0,08
UltraPure TBE Buffer, 10X (1L)	17,66	1000	0,02	1	0,02
SYBR Safe DNA Gel Stain (400µl)	70,27	400	0,18	1	0,18
GeneRuler 1kb DNA Ladder (2mL)	42,01	3300	0,01	1	0,01
6X Orange DNA Loading Dye (5mL)	28,51	330	0,09	1	0,09
**PCR product purification**					
ExoSAP-IT Express PCR Product Cleanup Reagent	237,90	480	0,50	1	0,50
**Sequencing and analysis**					
BigDye Terminator v3.1 Cycle Sequencing Kit (100 reactions)	1168,20	250	0,58	1	4,67
BigDye XTerminator Purification Kit (1000 reactions)	1346,40	250	0,67	1	5,39
POP-7 Polymer (25mL)	1578,60	8000	0,20	8	1,58
3730 Running Buffer, 10X (500mL)	232,50	200	1,16	1	1,16
Sequencing Primers	13,33	11250	0,00	8	0,01
**Consumables**					
1000µl tips (960)	132,09	960	0,14	6	0,83
200µl tips (960)	168,21	960	0,18	34	5,96

20µl tips (960)	112,14	960	0,12	6	0,70
10µl tips (960)	168,28	960	0,18	64	11,22
MicroAmp 8-Tube Strip with Caps, 0.2mL	101,96	1000	0,10	4	0,41
1.5mL Nuclease-Free Eppendorf Tubes	63,13	500	0,13	6	0,76
50mL Falcon Tubes	132,47	25	5,30	1	5,30
MicroAmp Optical 96-Well Reaction Plates (10)	56,13	960	0,06	8	0,47
Adhesive PCR Plate Foils (100)	69,59	1000	0,07	1	0,07
**Total**					**$US48,52**

dNTP, deoxynucleoside triphosphate; g, gram; kb, kilobase; mL, millilitre; mM, millimolar; PCR, polymerase chain reaction; µl, microliter; µM, micromolar

Note: The exchange rate used was ~$1 US Dollar equivalent to ZAR15 South African Rands
